# Negative Effective Mass in Plasmonic Systems II: Elucidating the Optical and Acoustical Branches of Vibrations and the Possibility of Anti-Resonance Propagation

**DOI:** 10.3390/ma13163512

**Published:** 2020-08-09

**Authors:** Edward Bormashenko, Irina Legchenkova, Mark Frenkel

**Affiliations:** Department of Chemical Engineering, Ariel University, Ariel 407000, Israel; ilegchenkova@gmail.com (I.L.); markfr@ariel.ac.il (M.F.)

**Keywords:** metamaterials, negative effective mass, plasma oscillations, low frequency plasmons, optical and acoustical branches

## Abstract

We report the negative effective mass metamaterials based on the electro-mechanical coupling exploiting plasma oscillations of free electron gas. The negative mass appears as a result of the vibration of a metallic particle with a frequency *ω* which is close to the frequency of the plasma oscillations of the electron gas $m_{2}$, relative to the ionic lattice $m_{1}$. The plasma oscillations are represented with the elastic spring constant $k_{2}=\omega_{p}^{2}m_{2}$, where $\omega_{p}$ is the plasma frequency. Thus, the metallic particle vibrating with the external frequency *ω* is described by the effective mass $m_{eff}=m_{1}+\frac{m_{2}\omega_{p}^{2}}{\omega_{p}^{2}-\omega^{2}}$, which is negative when the frequency ω approaches $\omega_{p}$ from above. The idea is exemplified with two conducting metals, namely Au and Li embedded in various matrices. We treated a one-dimensional lattice built from the metallic micro-elements $m_{eff}$ connected by ideal springs with the elastic constant $k_{1}$ representing various media such as polydimethylsiloxane and soda-lime glass. The optical and acoustical branches of longitudinal modes propagating through the lattice are elucidated for various ratios $\frac{\omega_{1}}{\omega_{p}}$, where $\omega_{1}^{2}=\frac{k_{1}}{m_{1}}$ and $k_{1}$ represents the elastic properties of the medium. The 1D lattice, built from the thin metallic wires giving rise to low frequency plasmons, is treated. The possibility of the anti-resonant propagation, strengthening the effect of the negative mass occurring under *ω* = *ω*_p_ = *ω*_1_, is addressed.

## 1. Introduction

Metamaterials are artificial materials demonstrating properties that are not found in naturally occurring materials [1,2,3]. In metamaterials, the index of refraction and magnetic permittivity may be negative at certain frequencies. Moreover, they may be tuned in a broad range of values [4]. One of the most rapidly developed fields within the domain of metamaterials is the field of photonic band-gap crystals, which are multidimensional periodic structures with a period of order of the optical wavelengths [5,6,7]. The theory predicted the existence of a photonic bandgap (PBG), a frequency band of inhibited optical modes [5,6]. Analogously, acoustical band gap materials were predicted and manufactured [8,9,10,11]. In particular, resonance sonic crystals, based on the idea of localized resonant structures, that exhibit spectral gaps with a lattice constant two orders of magnitude smaller than the relevant wavelength are reported in the literature [10,11].

Acoustic metamaterials, in which both the effective density and bulk modulus are simultaneously negative, in the true and strict sense of an effective medium have been reported [12]. Acoustic metamaterials demonstrating a negative Poisson’s ratio [13] and negative elastic modulus were discussed [14]. Mechanical metamaterials exhibiting auxetic behaviors and negative compressibility were suggested [15]. Acoustic metamaterials demonstrate a potential to be perfect absorbers of mechanical vibrations [16] and also of materials enabling the focusing of ultrasound [17]. In our recent paper, we proposed the exploitation of the plasma oscillations of the electron gas for the development of the metamaterials with the negative effective mass (density) [18]. The plasma oscillations in this model are represented with the elastic spring [18]. The notion of the negative effective mass (density) acoustic metamaterials was introduced in Refs. [19,20,21]. We suggested the exploitation of the so-called plasma oscillations of the electron gas [22] for the development of the metamaterials with a negative effective mass or density [18]. Now we elucidate the structure of the optical and acoustical branches of elastic waves propagating in chain structures built of elements possessing a negative effective mass, exploiting the plasma oscillations in metal particles connected by ideal springs and representing elastic media.

## 2. Results and discussion

### 2.1. Propagation of Harmonic Waves in the 1D Lattice Comprising Negative Effective Mass Plasmonic Elements

**Figure 1:**
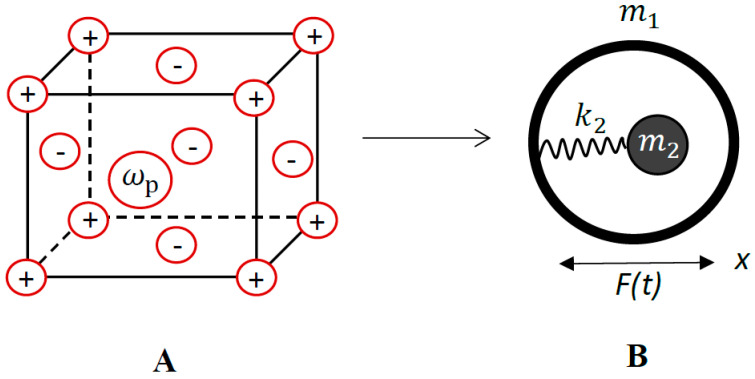
(**A**) Free electron gas is embedded in the ionic lattice; *ω_p_* is the electron plasma frequency (**B**). The equivalent mechanical scheme of the system (**A**). Core with mass *m*_2_ (free electron gas mass) is connected internally through the spring with $k_{2}=\omega_{p}^{2}m_{2}$ to a shell with mass *m*_1_ (ionic lattice mass). The system is subjected to the sinusoidal force $F\left(t\right)=\hat{F}sin\omega t$.

The mechanical model giving rise to the negative effective mass effect, introduced in Refs. [20,21,23] is depicted in Figure 1.

A core with mass $m_{2}$ is connected internally through the spring with the elastic constant $k_{2}$ to a shell with mass $m_{1}$. The system is submitted to the external sinusoidal force $F=\hat{F}sin\omega t$. If we solve the equations of motion for the masses $m_{1}$ and $m_{2}$ and replace the entire system with a single effective mass $m_{eff}$, we obtain [20,21,23] Equation (1):

(1) $$m_{eff}=m_{1}+\frac{m_{2}\omega_{0}^{2}}{\omega_{0}^{2}-\omega^{2}}$$

where $\omega_{0}=\sqrt{\frac{k_{2}}{m_{2}}}$. It is easily recognized that when the frequency ω approaches $\omega_{0}$ from above, the effective mass $m_{eff}$ will be negative [20,21,23]. In our recent paper we suggested the electro-mechanical, plasmonic analogy of the aforementioned model, giving rise to the negative effective mass [18]. Consider the cubic metal particle shown in Figure 1A, seen as an atomic lattice $m_{1}$ containing the Drude–Lorenz free electron gas possessing a total mass of $m_{2}=m_{e}nV$, where $m_{e}=9.1\times 10^{-31}$ kg is the mass of electron, *n* is the concentration (number density) of the electron gas and *V* is the volume of the particle [22,24,25]. Electron gas is free to oscillate with the plasma frequency $\omega_{p}=\sqrt{\frac{ne^{2}}{m_{e}\varepsilon_{0}}}$ [22,24,25]. Exposing the entire metal particle to the external sinusoidal force given by $F=\hat{F}sin\omega t$. The effective mechanical scheme of the metallic particle is shown in Figure 1B and it exactly coincides with that giving rise to the negative effective mass, supplied in this case by, Equation (2):

(2) $$m_{eff}=m_{1}+\frac{m_{2}\omega_{p}^{2}}{\omega_{p}^{2}-\omega^{2}}$$

where $m_{1}$ is the mass of the ionic lattice, $m_{2}$ is the total mass of the electron gas. It can be seen that it may be negative when the frequency ω approaches $\omega_{p}$ from above [18]. It was demonstrated that the effective dimensionless mass $\frac{m_{eff}}{m_{1}+m_{2}}≅\frac{m_{eff}}{m_{1}}$ is independent on the metallic particles’ size [18]. The results of calculations of the effective negative mass for Li and Au are supplied in Ref. [18] (the physical parameters of these metals are summarized in Table 1).

**Figure 2:**
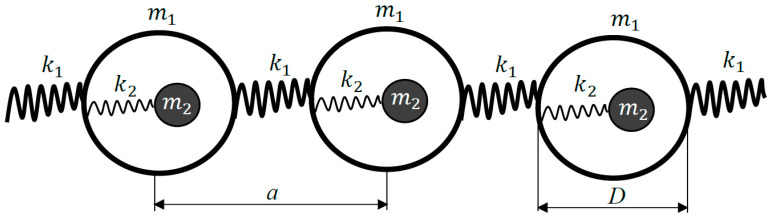
The mechanical scheme of the one-dimensional lattice is depicted.

Consider now the one-dimensional lattice built from the elements (cells) shown in Figure 1B and depicted in Figure 2. The 1D lattice is built up of identical elements possessing the effective negative masses $m_{eff}$ given by Equation (2) and connected by ideal springs with the elastic constant $k_{1}$; the separation between the elements *a* is constant, as shown in Figure 2.

Considering the propagation of harmonic waves (ω,q), Equation (3):

(3) $$u_{i}^{k+n}\left(x,t\right)={\hat{u}}_{0}e^{j\left(qx+nqa-\omega t\right)}$$

where $u_{i}^{k+n}\left(x,t\right)$ is the displacement of the mass *i* (i=1,2) in the k+n-cell, ${\hat{u}}_{0}$ is the complex wave amplitude, and *q* is the wave number [21]. The dispersion equation for the 1D lattice depicted in Figure 2 was derived in Ref. [21], Equation (4):

(4) $$m_{1}m_{2\;}\omega^{4}-\left[\left(m_{1}+m_{2}\right)k_{2}+2m_{2}k_{1}\left(1-cos\left(qa\right)\right)\right]\omega^{2}+2k_{1}k_{2}\left(1-cos\left(qa\right)\right)=0$$

Dividing Equation (4) by $m_{1}m_{2}$, and considering $\frac{m_{1}}{m_{2}}\gg 1$ (which is true for plasmonic systems, thus, we can neglect $m_{2}$ in the sum $m_{1}+m_{2})$ and $k_{2}=m_{2}\omega_{p}^{2}$, yields Equation (5):

(5) $$\omega^{4}-\left[\omega_{p}^{2}+2\frac{k_{1}}{m_{1}}\left(1-cos\left(qa\right)\right)\right]\omega^{2}+2\frac{k_{1}}{m_{1}}\omega_{p}^{2}\left[1-cos\left(qa\right)\right]=0$$

Denoting $\omega_{1}^{2}=\frac{k_{1}}{m_{1}}$ supplies in turn, Equation (6):

(6) $$\omega^{4}-\left[\omega_{p}^{2}+2\omega_{1}^{2}\left(1-cos\left(qa\right)\right)\right]\omega^{2}+2\omega_{1}^{2}\omega_{p}^{2}\left[1-cos\left(qa\right)\right]=0$$

Equation (6) yields the following exact solutions, Equations (7) and (8):

(7) $$\omega =\omega_{p}$$

(8) $$\omega =\omega_{1}\sqrt{2\left(1-cos\left(qa\right)\right)}$$

The solution of Equation (6) gives rise to the “acoustic” and “optical” branches of vibrations [26,27].

The solution supplied by Equation (7) is intrinsic to the plasma oscillations of the electron gas and corresponds to the absence of dispersion within the optical branch of vibrations; whereas, the solution supplied by Equation (8) corresponds to the well-known dispersion relation inherent to the propagation of longitudinal acoustic waves spreading within a homogeneous 1D lattice, possessing the lattice constant *a* [27]. In the limiting case of qa→0, we obtain the non-dispersion propagation $\omega =\omega_{1}qa$ corresponding to the continuous string, possessing the highest eigenfrequency of $\omega =\omega_{1}$. The degenerated double-resonance propagation occurs when *ω* = *ω*_p_ = *ω*_1_ takes place. This propagation corresponds to the so-called anti-resonance, when the amplitude of vibration of the mass $m_{1}$ is minimal and in the limiting case even equals zero [28,29]. When the anti-resonance *ω* = *ω*_p_ = *ω*_1_ condition is fulfilled, all of the energy is transferred to the mass $m_{2}$, thus strengthening the effect of the negative mass.

**Figure 3:**
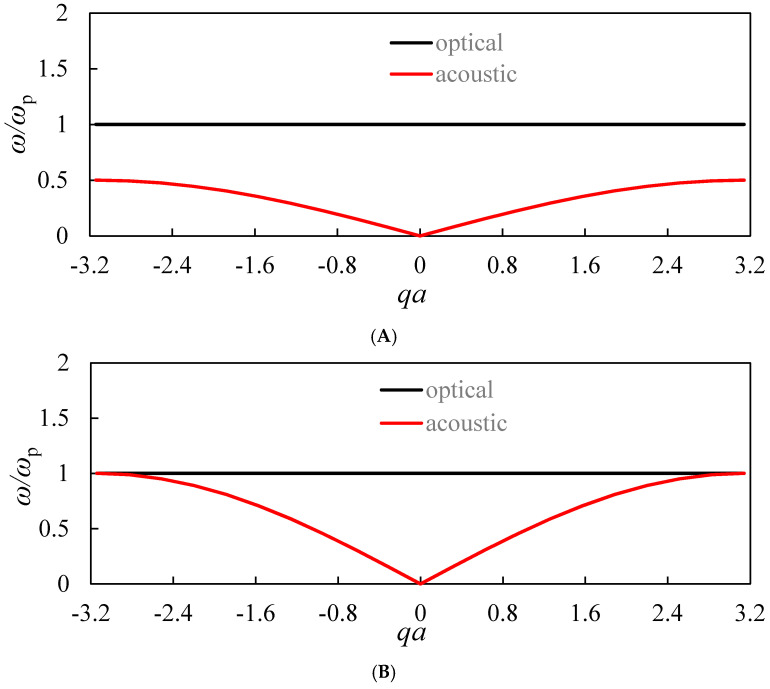

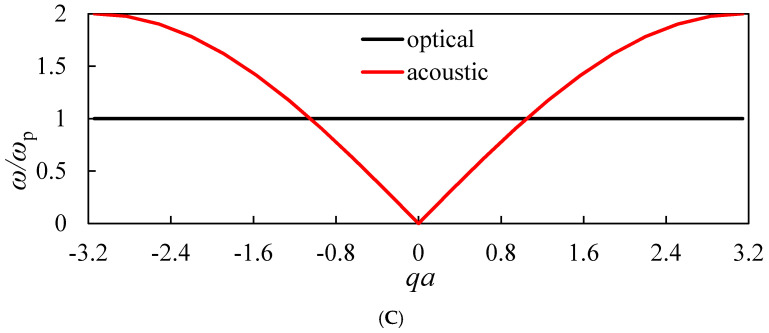
Optical and acoustic branches of longitudinal modes calculated for different ratios $\frac{\omega_{1}}{\omega_{p}}$. (**A**) $\frac{\omega_{1}}{\omega_{p}}$ = 0.25; (**B**) $\frac{\omega_{1}}{\omega_{p}}$ = 0.5; (**C**) $\frac{\omega_{1}}{\omega_{p}}$ = 1.

The “optical” and “acoustical” branches of longitudinal modes propagation in the 1D lattice, depicted in Figure 2 for various $\frac{\omega_{1}}{\omega_{p}}$ ratios, are shown in Figure 3A–C [26,27].

It is recognized from Figure 3, that the relative location and configuration of the optical and acoustical branches depends strongly on the ratio $\frac{\omega_{1}}{\omega_{p}}$. The optical and acoustical branches may be separated by the frequency (energy) gap, as shown in Figure 3A. The configurations of optical and acoustical branches at which this gap is zero are also possible, as shown in Figure 3B,C. It is noteworthy that the optical and acoustical branches may intersect, as depicted in Figure 3C.

In order to exemplify the suggested metamaterial, we considered the 1D lattice of spherical Li and Au particles, dispersed in the polymer (polydimethylsiloxane) and soda-lime glass matrices. The values of the spring stiffness *k*_1_ [30] and frequencies $\omega_{1}$ (representing the elastic media) calculated for various diameters of the metallic particles ($D≅10^{-7}-10^{-6}\;m$) and lattice constants ($a≅1.5\times 10^{-7}-1.5\times 10^{-6}\;m$) are summarized in Table 2.

It is clearly recognized from the numerical data supplied in Table 1 and Table 2, that for the suggested composite metamaterials takes place the inequality *ω_p_* >> *ω*_1_. Thus, the relative location of the acoustic and optical branches of modes, resulting in the formation of the band gap, depicted in Figure 3A, necessarily occurs. The situations presented in Figure 3B,C demand an essential decrease in the plasma frequency, which is possible in the metamaterials, addressed in the following section.

### 2.2. Propagation of Harmonic Waves in the Metallic Mesostructures Demonstrating the Effect of Negative Effective Mass

**Figure 4:**
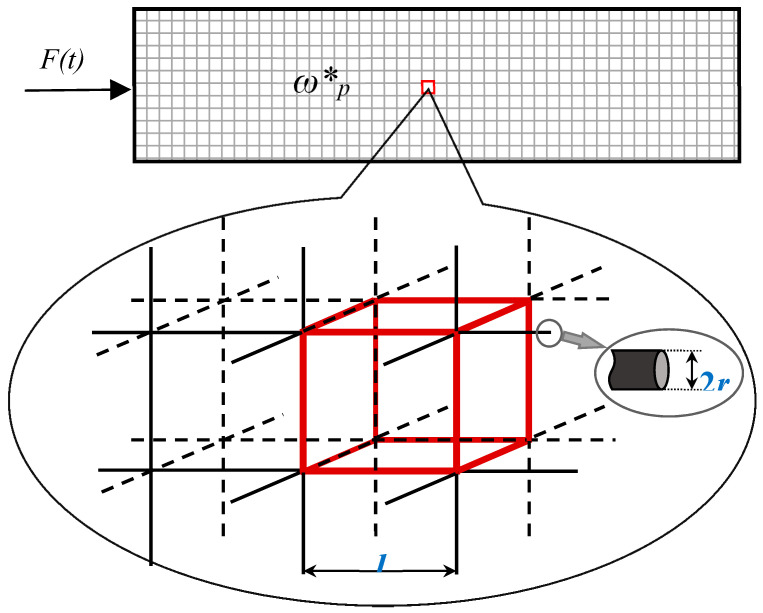
Metallic wires with a radius of *r* arranged in a simple cubic lattice with the lattice constant of *l*. The lattice is subjected to the axial sinusoidal force $F\left(t\right)=\hat{F}sin\omega t$

The plasma oscillations shown in Figure 1 will demonstrate the negative mass in the vicinity of the plasma frequency which is in the order of magnitude of $\omega_{p}≅10^{16}\;\mathrm{rad}/s$, which is very high. However, this frequency may be strongly decreased for the mesostructures built from thin metallic wires, as demonstrated in Ref. [31]. Depression of the plasma frequency into the far infrared and even GHz band becomes possible due to the mutual inductance appearing in the periodic arrays built of thin metallic wires arranged in a simple cubic lattice, joined at the corners of the lattice [31], as depicted in Figure 4.

Consider the longitudinal acoustic modes propagating along such a lattice. For the sake of simplification, we replaced the 3D lattice with the 1D lattice, shown in Figure 2. The effective (pseudo) density of electrons in the metamaterial lattice shown in Figure 4 is given by [31], Equation (9):

(9) $$\tilde{n}≅\pi n\frac{r^{2}}{l^{2}}$$

where *l* is the lattice constant, *r* is the radius of the wire, and *n* is the concentration of the free electron gas supplied in Table 1 for Li and Au. The pseudo-mass of electrons in such matrices is given by [31], Equation (10):

(10) $$\tilde{m}=\frac{\mu_{0}r^{2}e^{2}n}{2}ln\frac{l}{r}$$

where $\mu_{0}$ is the magnetic permittivity of the vacuum. The value $\tilde{m}$ expressed by Equation (10) is called in Ref. [31] as the “effective mass”; however, in our paper, the notion of the “effective mass” is already ascribed to the mass of the vibrated element, given by Equation (1). Thus, we call the value expressed by Equation (10) the “pseudo-mass”, and the effective density of electrons expressed by Equation (9) we label as the “pseudo-density”. Assuming $r=1.0\times 10^{-6}\;m;l=5.0\times 10^{-3}\;m$ (which is typical for metamaterials) enables the calculation of the effective pseudo-plasma frequencies $\omega_{p}^{*}$ for Au and Li according to Equation (11) (Ref. [31]):

(11) $$\omega_{p}^{*}=\sqrt{\frac{\tilde{n}e^{2}}{\varepsilon_{0}\tilde{m}}}=\sqrt{\frac{2\pi c_{0}^{2}}{l^{2}ln(l/r)}}$$

where $c_{0}≅3.0\times 10^{8\;}\frac{m}{s}$ is the speed of light in the vacuum. Substituting the aforementioned numerical parameters yield the effective plasma frequencies of the lattices built from Au and Li wires $\omega_{p}^{*Au}=\omega_{p}^{*Li}=8.2\;\mathrm{rad}/s$, which are already comparable with the frequencies attainable by the modern piezoelectric devices [32,33].

The relative location of the optical and acoustical branches of the longitudinal modes’ propagation in the 1D meta-lattice, depicted in Figure 4, is similar to that shown in Figure 3. However, contrastingly to the situation addressed in the previous section, the approximate equality $\omega_{p}≅\omega_{1}$ becomes attainable under the reasonable choice of the geometrical parameters *l* and *r*. Thus, the anti-resonant propagation, strengthening the effect of the negative mass under *ω* = *ω*_p_ = *ω*_1_ becomes possible [28,29].

Again, the configurations of the optical and acoustic branches separated and non-separated by the frequency (energy) gap are possible, as illustrated in Figure 3. It should be emphasized that the ensembles of metallic wires, shown schematically in Figure 4, will not demonstrate simultaneously the negative mass (density) and the negative refraction effects [34]. This is due to the fact that the negative refraction becomes possible below the plasma frequency $\omega_{p}$ [34]; contrastingly, the effect of the negative mass in our model emerges when the frequency ω approaches $\omega_{p}$ from above; therefore, it remains a challenge to create a material that simultaneously presents a negative density and a negative dielectric constant.

## 3. Conclusions

We address the propagation of harmonic longitudinal acoustic waves through a 1D lattice, demonstrating the effect of the negative mass arising from the plasma oscillations of the electron gas relatively to the atomic lattice. The effect takes place when a metallic particle vibrates with the external frequency ω approaching the plasma frequency $\omega_{p}=\sqrt{\frac{ne^{2}}{m_{e}\varepsilon_{0}\;}}$ from above. In this case, the effective mass of the metallic particle $m_{eff}=m_{1}+\frac{m_{2}\omega_{p}^{2}}{\omega_{p}^{2}-\omega^{2}}$, where $m_{1}$ is the mass of the ionic lattice, and $m_{2}$ is the mass of the electron gas, becomes negative [12,13,15,18,21].

The plasma oscillations may be phenomenologically represented with the ideal elastic spring constant $k_{2}=\omega_{p}^{2}m_{2}$. In this paper, a one-dimensional lattice built of identical metallic (Li and Au) elements, with effective mass $m_{eff}$ connected by ideal springs with the elastic constant $k_{1}$ that allows electromechanical coupling, is addressed. A model of metamaterials built of Li and Au micro-particles embedded into polymer and glass matrices, represented by the referred ideal elastic springs with the constant $k_{1}$, is also considered. Thus, dispersion relationships are clarified in the case where $\frac{m_{1}}{m_{2}}\gg 1$. The configurations of the optical and acoustical branches of the longitudinal modes propagating through the 1D lattice arising from the various ratios $\frac{\omega_{1}}{\omega_{p}}$ are explored [26,27]. The relative location and configuration of the optical and acoustical branches depend strongly on the ratio $\frac{\omega_{1}}{\omega_{p}}$. The optical and acoustical branches may be separated by the frequency (energy) gap. We also deal with the possibility of anti-resonant wave propagation when $\omega =\omega_{p}=\omega_{1}=\sqrt{\frac{k_{1}}{m_{1}}}$ takes place.

The effects due to the negative effective mass become possible in the nearest vicinity of the plasma frequencies, namely $\omega_{p}\sim 10^{16}\mathrm{rad}/s$, which is characteristic of a typical metal. The plasma frequency may be decreased markedly for the low frequency plasmons predicted for the metallic mesostructures [31], allowing the creation of metamaterials that demonstrate negative effective densities. This shows that the possible configurations of the optical and acoustic branches are separated and non-separated by the frequency (energy) gap. Again, the anti-resonant propagation, strengthening the effect of the negative mass under *ω* = *ω*_p_ = *ω*_1_ is feasible [28,29]. It should be emphasized that our paper neglects completely the effects of losses inevitable in plasmas, among which the Landau damping and radiation losses may affect the derived results [35,36]. We addressed in our paper 1D plasma oscillations giving rise to the effect of negative mass in metals; however, plasma oscillations are also observed in 2D systems [37,38,39,40], such as graphene, where losses may be relatively small.

## Figures and Tables

**Table 1:** Properties of the metals used in the calculations.

Metal	*ρ*, kg/m^3^	$n_{e},$ m^−3^	*p*, Hz
Li	530	4.7 × 10^28^	1.0 × 10^16^
Au	19,300	5.9 × 10^28^	1.3 × 10^16^

**Table 2:** Dimensions of spherical metallic particles and physical properties of the matrix materials used in the calculations.

Metal	*D*, m	a, m	*m*_1_, kg	*m*_2_, kg	*k*_1_, N/m (PDMS)	*k*_1_, N/m (Glass)	*k*_2_, N/m (Plasma)	*ω*_1_, Hz (PDMS)	*ω*_1_, Hz (Glass)
Au	1 × 10^−6^	1.5 × 10^−6^	1.01 × 10^−14^	2.81 × 10^−20^	1.18	1.1 × 10^5^	3.65 × 10^−4^	1.72 × 10^6^	5.25 × 10^8^
Li	1 × 10^−6^	1.5 × 10^−6^	2.77 × 10^−16^	2.24 × 10^−20^	1.18	1.1 × 10^5^	2.24 × 10^−4^	1.04 × 10^7^	3.17 × 10^9^
Au	5 × 10^−7^	7.5 × 10^−7^	1.26 × 10^−15^	3.51 × 10^−21^	0.59	5.5 × 10^4^	4.57 × 10^−5^	3.44 × 10^6^	1.05 × 10^9^
Li	5 × 10^-7^	7.5 × 10^−7^	3.46 × 10^−17^	2.80 × 10^−21^	0.59	5.5 × 10^4^	2.80 × 10^−5^	2.08 × 10^7^	6.34 × 10^9^
Au	1 × 10^−7^	1.5 × 10^−7^	1.01 × 10^−17^	2.81 × 10^−23^	0.12	1.1 × 10^4^	3.65 × 10^−7^	1.72 × 10^7^	5.25 × 10^9^
Li	1 × 10^−7^	1.5 × 10^−7^	2.77 × 10^−19^	2.24 × 10^−23^	0.12	1.1 × 10^4^	2.24 × 10^−7^	1.04 × 10^8^	3.2 × 10^10^

*D*—diameter of the spherical metallic particle (Figure 2); *a*—lattice constant.
